# Long-term upper-extremity prosthetic control using regenerative peripheral nerve interfaces and implanted EMG electrodes

**DOI:** 10.1088/1741-2552/accb0c

**Published:** 2023-04-25

**Authors:** Philip P Vu, Alex K Vaskov, Christina Lee, Ritvik R Jillala, Dylan M Wallace, Alicia J Davis, Theodore A Kung, Stephen W P Kemp, Deanna H Gates, Cynthia A Chestek, Paul S Cederna

**Affiliations:** 1 Department of Biomedical Engineering, University of Michigan, Ann Arbor, MI 48109, United States of America; 2 Section of Plastic Surgery, University of Michigan, Ann Arbor, MI 48109, United States of America; 3 Robotics Institute, University of Michigan, Ann Arbor, MI 48109, United States of America; 4 School of Kinesiology, University of Michigan, Ann Arbor, MI 48109, United States of America; 5 Department of Electrical Engineering and Computer Science, University of Michigan, Ann Arbor, MI 48109, United States of America; 6 Neuroscience Graduate Program, University of Michigan, Ann Arbor, MI 48109, United States of America; 7 University of Michigan Hospital Orthotics & Prosthetics Center Ann Arbor, Ann Arbor, MI 48109, United States of America

**Keywords:** neuroprosthetics, peripheral nerve regeneration, myoelectric control, pattern recognition

## Abstract

*Objective.* Extracting signals directly from the motor system poses challenges in obtaining both high amplitude and sustainable signals for upper-limb neuroprosthetic control. To translate neural interfaces into the clinical space, these interfaces must provide consistent signals and prosthetic performance. *Approach.* Previously, we have demonstrated that the Regenerative Peripheral Nerve Interface (RPNI) is a biologically stable, bioamplifier of efferent motor action potentials. Here, we assessed the signal reliability from electrodes surgically implanted in RPNIs and residual innervated muscles in humans for long-term prosthetic control. *Main results.* RPNI signal quality, measured as signal-to-noise ratio, remained greater than 15 for up to 276 and 1054 d in participant 1 (P1), and participant 2 (P2), respectively. Electromyography from both RPNIs and residual muscles was used to decode finger and grasp movements. Though signal amplitude varied between sessions, P2 maintained real-time prosthetic performance above 94% accuracy for 604 d without recalibration. Additionally, P2 completed a real-world multi-sequence coffee task with 99% accuracy for 611 d without recalibration. *Significance.* This study demonstrates the potential of RPNIs and implanted EMG electrodes as a long-term interface for enhanced prosthetic control.

## 1. Introduction

Major upper-limb amputation is a devastating injury, leading not only to the inability to perform tasks and activities of daily living, but also to concomitant issues, such as depression, compensatory overuse injuries, and loss or diminished employment [1–3]. In the United States alone, approximately 41 000 people live with major upper-limb amputations [4], and global prevalence is estimated in the millions [5]. Many advancements in myoelectric prosthetic arm systems have allowed control of multiple degrees of freedom (DoFs), which can include individual finger, wrist, and elbow control [6–9]. However, despite these technological advancements, end-users do not have accurate and reliable control over these additional DoFs [10, 11]. In fact, 10%–25% of people with upper-limb amputations choose not to use a prosthesis, and those who do, around 50% use a myoelectric prosthesis [12–16].

Currently, the clinical standard of control for upper-limb myoelectric prosthetic users is direct dual-site control, in which available agonist–antagonist muscle pairs modulate a single degree of freedom of a prosthetic hand (e.g. hand open/close) [17]. A residual muscle’s electromyogram (EMG) signal controls the direction of the motor on the prosthetic joint, while their signal amplitude proportionally controls the motor speed. Clinicians typically place a pair of electrodes on the surface of the residual limb to obtain these surface EMG signals [17, 18]. However, the agonist–antagonist muscle pairs’ physiological function may not correspond to the same function on the prosthesis. For example, the wrist flexor and extensor muscle pair would be used to control hand open/close for a person with a transradial amputation. This control paradigm becomes unintuitive, cumbersome, and limits prosthetic function for the end-users, which negatively impacts their functional expectations and can contribute to prosthesis abandonment [15].

To command more DoFs, commercial and research groups have implemented grip selection mechanisms to switch between arm and hand postures. One of the more commonly employed approaches uses specific surface EMG patterns, such as co-contracting of a muscle pair, to trigger a transition from one grip to another (e.g. switch hand open/close to wrist supination/pronation) [19]. In other approaches, the person uses specific body movements (i.e. the forearm or foot) attached with inertial measurement units to trigger this transition [20, 21]. Unfortunately, these existing control methods remain unintuitive and time-consuming during activities of daily living and do not provide naturalistic function [21–24].

Pattern recognition systems have been developed to provide more intuitive and consistent control of grip switching [25–31]. In this approach, multiple surface recording electrodes (typically eight sites) capture the surface EMG of residual innervated muscles or reinnervated muscles created from targeted muscle reinnervation (TMR) [32], which can be classified to a specific hand posture or grasp [33]. TMR surgically reroutes transected nerves to reinnervate existing muscle to regain lost motor function. If unique muscle activity patterns correlate with a specific grasp, then this approach can provide prosthesis users with more intuitive control of multiple grips. Comparison studies have demonstrated that pattern recognition systems can outperform direct control in several functional assessments [29, 34]. However, despite the improved prosthetic performance with pattern recognition, the limitations associated with surface EMG still affect controller reliability. Donning and doffing the prostheses often requires a system recalibration and even changes in arm position during use have been shown to reduce controller accuracy [35–38]. The number of functional grips reliably achieved in real-world scenarios is 2 or 3 compared to the 9 or 12 reported in laboratory settings [33, 39]. With inconsistent control, users are forced to recalibrate the system frequently, which can cause end-user frustration [34].

To provide more intuitive and consistent functional control of multi-articulated prosthetic hands, many invasive approaches have been employed to overcome some of the shortcomings of surface EMG [40–45]. Electrode recordings of efferent motor action potentials directly from the peripheral nerve have been able to demonstrate control of multiple DoFs of individuated fingers proportionally and simultaneously [45–48]. However, it has been very challenging to achieve long term stability of neural recordings with this approach. For instance, the longest demonstrated stable peripheral nerve interface in a person with limb loss was 16 months with only 9% of the electrode sites being active and functional at this time point [49]. Unfortunately, other direct nerve recording approaches have not reported data longer than 2–8 months following implantation and face similar challenges in maintaining consistent functional sites [50]. Furthermore, each of these peripheral nerve interfaces have limitations regarding nerve specificity, tissue injury, axonal degeneration, or scar tissue formation associated with a chronic indwelling foreign body response.

Alternatively, surgically implanting intramuscular electrodes in residual innervated muscles and muscles that underwent TMR can achieve more robust electrode recordings of prosthetic control signals [44, 46, 51, 52]. Studies have demonstrated improved control accuracy and reduced movement variability using intramuscular or epimysial EMG recordings, independent of changes to the control strategy [43, 44, 52–54]. Specifically, people with above elbow amputations implanted with a percutaneous bone-anchored interface (osseointegration) and TMR had a functional prosthesis after 3–7 years of use with one participant returning to full-time employment [53]. People with transradial amputations demonstrated stable three-DoF prosthetic control in a virtual space up to 10 months without recalibration using percutaneous intramuscular electrodes in residual innervated muscles [54]. These studies have shown promising long-term use of implanted electrodes. However, osseointegration remains limited in the number of electrodes that can be fed through the bone-anchored interface (up to four bipolar electrodes), which limits the number of acquired control signals and thus, prosthetic functionality [43]. Lukyanenko *et al* demonstrated reliable control in the virtual space, but to show full efficacy, studies need to evaluate prosthetic performance with a physical real-world task for clinical translation.

To provide more independent control sites and capture lost efferent motor activity from severed peripheral nerves, our group has developed the Regenerative Peripheral Nerve Interface (RPNI) as a biologic interface to increase prosthetic function [55–58]. The RPNI is created by surgically implanting the distal end of a transected nerve into an autogenous free muscle graft [59]. The nerve undergoes axonal sprouting, elongation, and reinnervation of the free muscle graft to create the RPNI. This process occurs within 8 weeks following implantation [55, 58]. Efferent motor action potentials cause the RPNI to contract evoking relatively large EMG signals with a high signal-to-noise ratio (SNR) even when RPNIs are created on individual nerve fascicles. In essence, the RPNIs act as a bioamplifier of the efferent motor action potentials [55, 56]. Extensive and robust rodent, non-human primate, and human data have demonstrated the feasibility, and high signal fidelity of the RPNIs [55, 56, 60–63].

In previous work, we have shown that RPNIs have high SNR at a few time points, up to 2–3 years after RPNI creation [56]. Here, we conducted a systematic analysis on the quality of recorded signals to understand the long-term signal reliability, measuring at monthly intervals (outside of COVID-19 pauses) up to 1054 d and 276 d after electrode implantation in two individuals with limb loss. We show that both residual muscle and RPNI signals do not significantly decrease over time, despite day-to-day variability. We then evaluated the long-term prosthetic performance in one participant, training a decoder on a single day of recordings and reusing the decoder without recalibration up to 604 d. Specifically, we decoded four different hand postures across four different arm postures in online experiments and showed a median performance of 95.9%. Finally, we demonstrated the feasibility of using our four-class decoder in a representative activity of daily living in one individual for 611 d without recalibration. Overall, we found that intramuscular signals clearly generate reliable prosthesis function without the need to recalibrate as opposed to surface electrode signals [29], and RPNIs can consistently generate SNRs greater than direct nerve recordings [45, 46, 50].

## 2. Methods

The Institutional Review Board at the University of Michigan approved this study (HUM00124839), and each participant provided written and informed consent. Detailed descriptions of the implantations, signal processing, and decoding algorithm were described previously [56, 63]. The authors have confirmed that any identifiable participants in this study have given their consent for publication.

### 2.1. Electrode implant

For clarity, participant 1 (P1) and participant 2 (P2), who had transradial amputations, underwent RPNI surgery for the treatment of their neuroma pain and phantom pain. One-year post-RPNI surgery, eight indwelling bipolar electrodes were implanted to record EMG from the RPNIs and several residual muscles. P1 had one RPNI created on each median and ulnar nerve, whereas P2 had one RPNI created on the median nerve and two RPNIs created on the divided ulnar nerve. Electrodes were placed in the following residual muscles: flexor pollicis longus, flexor digitorum profundus (FDP) index finger, FDP small finger, flexor carpi radialis (FCR), extensor digitorum communis (EDC), and extensor pollicis longus (EPL). P2 received electrodes in all residual muscles mentioned above except for FDP small finger to accommodate the additional ulnar RPNI.

### 2.2. Signal processing

Percutaneous connectors were attached to a neural signal processor (NeuroPort, Blackrock Microsystems), which recorded EMG signals at 30 ksps and filtered them between 3 and 7000 Hz (unity gain) for offline analysis. A Matlab target xPC (Mathworks) further filtered the EMG from 100 to 500 Hz, down-sampled the recording to 1 kSps, and decoded EMG into movement commands. EMG SNR from each electrode channel was calculated by taking the root mean square (RMS) of the EMG during volitional phantom finger movements and dividing by the RMS of the electrode’s noise floor seen during rest. For P1 and P2, 12 sessions (across 1 year) and 27 sessions (across 3 years) were analyzed for signal quality and SNR.

### 2.3. Online decoder analysis across multiple arm postures

**Figure 1.**
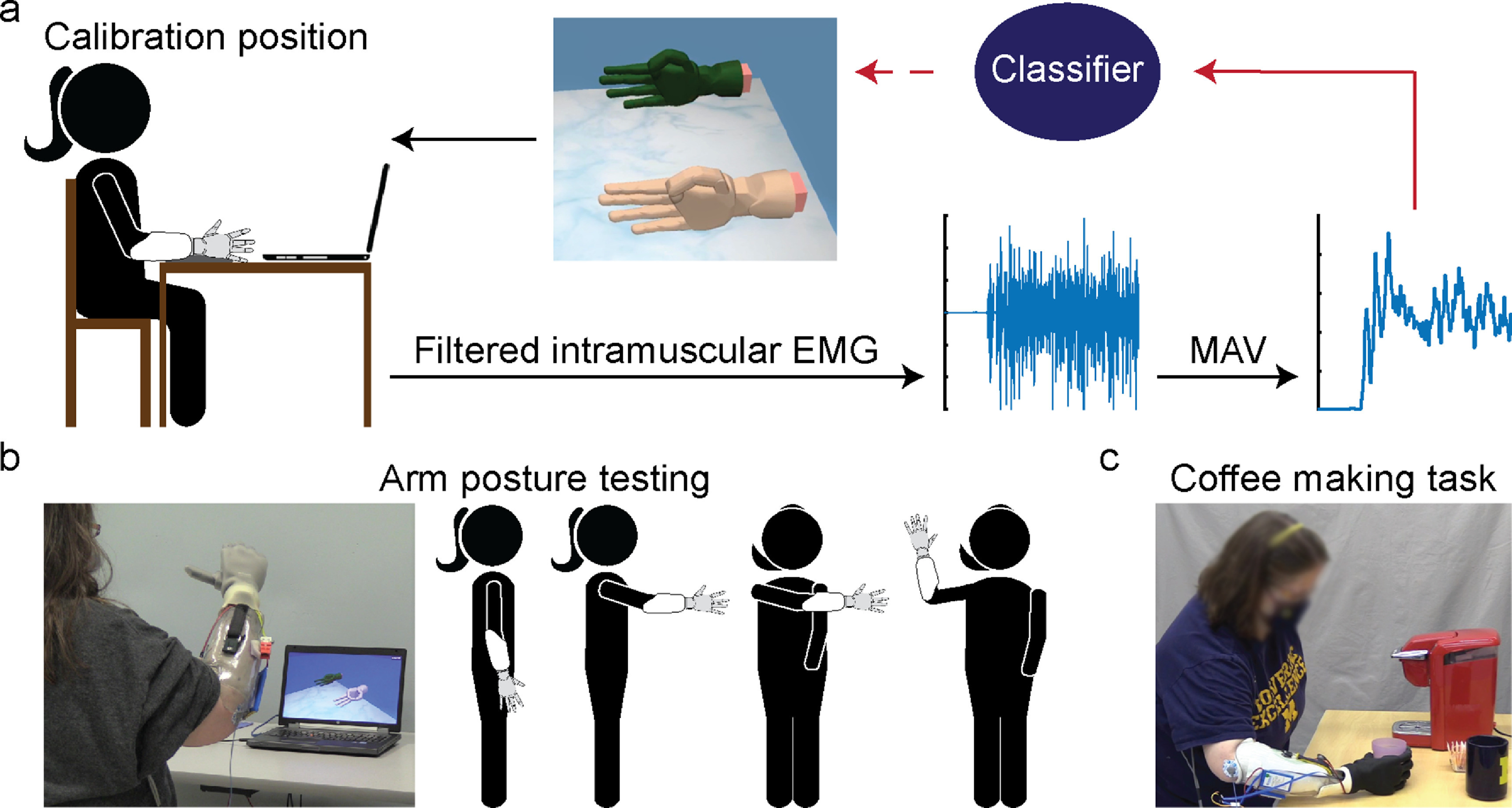
Experiment set-up and task description. (a) Participants mimicked a virtual hand with their phantom limb to collect intramuscular EMG for offline analyses and decoder calibration (solid red arrow). P2 controlled a second virtual hand to match cued postures during online decoding sessions (dashed red arrow). (b) To quantify decoding performance over time, P2 completed the virtual task in four static arm positions, with her inactive prosthesis donned to simulate weight effects. (c) P2 then used her prosthesis to complete a multi-grasp coffee making task.

P2 completed 16 experiment sessions over 604 d to assess the stability of a decoder performance over time. P1 also completed real-time control experiments in a previous study, often using weeks-old calibration data [63]. However, he withdrew from the clinical trial before the structured protocol to assess decoder stability was developed for this study. A Hidden Markov Model-Naïve Bayes (HMM-NB) classifier was trained using six channels (median RPNI, ulnar RPNI 1, ulnar RPNI 2, FDL, FDP index, EDC) to distinguish four functional grips: rest, fist, pinch, and point. FCR and EPL channels were excluded because they did not add information to the four movements. P2 completed one calibration session in a neutral arm position (arm supported on table) to train the HMM-NB, during which she mimicked the virtual display with her phantom hand for five repetitions of each movement (figure 1(a)). The Mean-Absolute-Value (MAV) time domain feature [25] was extracted from each channel in 50 ms non-overlapping time bins during each repetition. The calibration procedure and model training took less than 5 min to complete. The trained HMM-NB model parameters were fixed and reused for subsequent decoding sessions.

Briefly, the HMM-NB models dynamic EMG patterns evoked during finger movements as a series of latent states. The HMM-NB can leverage the high-resolution signals from intramuscular electrodes to accurately decode movements with faster processing windows than similar single-state models. Implementation of the HMM used here is detailed in previous work [63]. Decoder performance was quantified using a real-time posture matching discrete task in a virtual environment. A virtual cued hand was displayed, while the participant controlled a second virtual hand to match the cued grip. In these sessions, performance was measured in four arm positions detailed below (figure 1(b)):

*Arm at side*: arm relaxed in a natural position parallel to trunk;

*Arm in front*: shoulder flexed 50°, elbow flexed 130°;

*Arm across*: forearm parallel to floor, across chest such that the prostheses fingers extended 6” beyond the participant’s midline;

*Arm raised*: shoulder flexed 80°, elbow flexed 100°.

P2 used the HMM-NB to directly switch between the three postures and rest. The virtual task required her to hold a cued posture for 1 s continuously, with a timeout period of 5 s, before switching to a new pseudo-randomly cued posture. In each decoding session, she completed 20–30 trials of the virtual task in each arm position, with approximately 3 min of rest in between positions. Researchers used a goniometer to confirm her shoulder and elbow flexion angles for each arm position. Decoder performance was quantified by measuring transition errors between the start of EMG onset and the end of each trial. A transition error was counted if the decoder predicted any grip outside the cued grip during any 50 ms timestep. Decoder accuracy accounts for both the occurrence and duration of transition errors and was calculated as a percentage of correctly classified timesteps out of the total timesteps as follows:

\begin{equation*}{A_c} = \frac{{\mathop \sum \nolimits_{i \in {T_c}} \left[ {{x_i} = c} \right]}}{{n\left( {{T_c}} \right)}},\end{equation*}

where *A_c_* is the accuracy for movement *c, x_i_* is the logged decoder output at timestep *i*, and *T* is the set of timesteps analyzed.

To visualize the EMG features, infomax independent component analysis (ICA) with dimensionality reduction (principal component analysis (PCA)) was used to decompose the six channels into 2D space. The ICA-PCA technique was performed using the function runica() in the EEGLAB package [64, 65] (v4.5b; http://sccn.ucsd.edu/eeglab/).

### 2.4. Coffee making task

P2 also completed a bilateral Coffee Task that required use of all four functional grasps mentioned above using an extra small iLimb Quantum™ (Ossur, Reykjavik, Iceland). During the Coffee Task, she used a cup with water (simulated with beads), coffee pod, sugar, and a coffee brewer (Keurig™ mini (Reading, MA)) to simulate brewing a cup of coffee (figure 1(c)). The participant was asked to complete the full task continuously to quantify completion time. She also repeated the task in five segments, in which each segment measured the accuracy of transitioning into a specified grip (e.g. make a fist to pick up the cup with water). P2 repeated each segment five times across five trials with a total maximum number of possible transition errors of 125 (5 segments * 5 repetitions * 5 trials).

P2’s controller for the coffee task had an additional hand open movement (hand abduction) that allowed her to open the hand and then switch to a new grip. A grip selection filter and proportional control strategy were adapted from previous work [28, 66]. Designed to prevent sudden movements, which could occur with a discrete controller, the grip selection filter had a 250 ms threshold to actuate a new grip and the proportional controller attenuated her proportional control signal with a 500 ms velocity ramp. Calibration data for the hand open movement was also collected on the same calibration day as the four grips mentioned above. The coffee task was completed 590 and 611 d post-decoder training. In our previous study, P2 was able to use the three-grasp HMM, without an active open, to perform a simple object movement task. However, we concluded that a proportional adjustment of grip aperture would provide better control for a wider range of activities [63].

### 2.5. Offline decoder analysis of nine movements

The same calibration method and MAV feature extraction was used to collect data for the nine-movement offline analysis for five sessions over a span of 276 d for P1 and seven sessions over 463 d for P2. In addition to the HMM-NB, single-state Naïve Bayes (NB) and linear discriminant analysis (LDA) classifiers were trained with Matlab 2021a functions to predict rest, thumb, index, middle, ring, small finger flexion, wrist flexion, finger abduction, and finger adduction. Decoders were trained on the first day of collected data and not updated for subsequent sessions. Accuracy on the first day was measured using leave-one-out cross validation. Decoder accuracy was calculated on a per-trial basis by predicting movement from trial-averaged EMG for NB and LDA, and the mode of the HMM-NB output. This method attempts to ignore transient errors and was chosen to measure algorithm performance in an open loop setting.

### 2.6. Offline analysis of RPNI contributions to movement decoding

To highlight the contribution of RPNIs to movement decoding, a post-hoc analysis compared the performance of movement classifiers with input from only residual muscles to classifiers with input from residual muscles and RPNIs for both P1 and P2. In an offline analysis, LDA classifiers were trained and tested on multiple sessions for three movement sets: the four functional grips used in the arm posture test (*n* = 3 sessions across 173 d for P1, *n* = 4 sessions across 288 d for P2), the above nine movement analysis, and six intrinsic movements (*n* = 4 sessions across 212 d for P1, *n* = 7 sessions across 463 d for P2). The third movement set focused on distinguishing thumb and finger movements controlled by intrinsic hand muscles (thumb opposition, finger abduction, finger adduction) from thumb flexion, index flexion, and rest. Decoders were retrained for each session, accuracy was calculated as described above, then averaged across all sessions. RPNIs were inferred to be the most valuable for predicting the movements that were most negatively affected by their removal as a decoder input.

### 2.7. Statistical analysis

Trends in decoder performance and SNR were assessed with a linear regression model. A non-zero slope, i.e. change in SNR or decoder performance over time, was evaluated with an *F*-test. If a significant change was detected, the sign of the slope then determined an increasing or decreasing linear trend over time. Online decoding accuracy between arm positions used a one-way ANOVA with Bonferroni correction for multiple comparisons. Offline comparison of decoding algorithms used a Wilcoxon rank sum test. All statistical comparisons were analyzed with a significance level of *α* = 0.05.

## 3. Results

### 3.1. RPNIs produce large signals for long time periods

**Figure 2.**
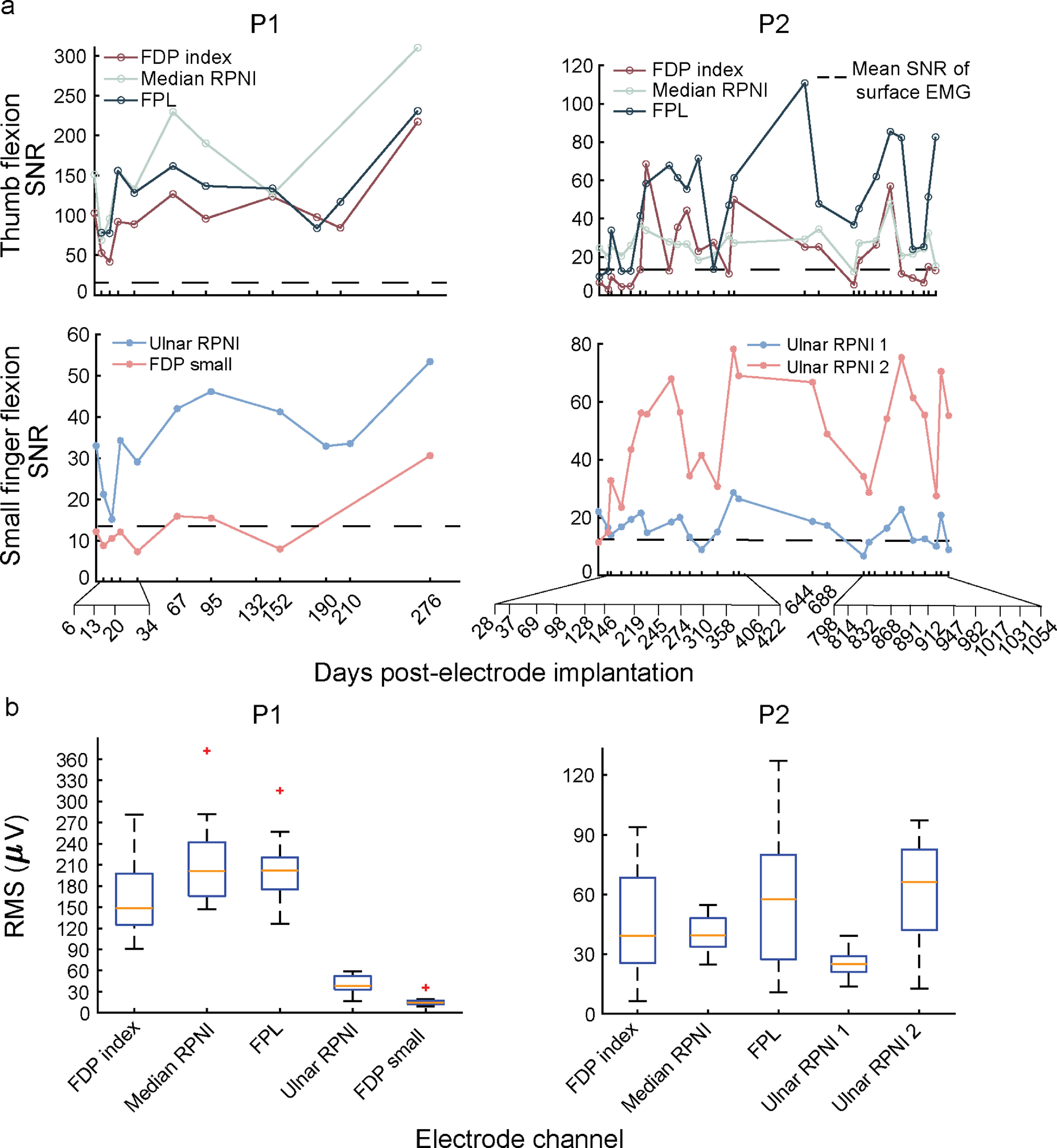
Measured signal-to-noise ratios (SNRs) over time. (a) Measured SNRs for P1 and P2 during volitional phantom movements of thumb and small finger flexion. SNRs remained high for both RPNIs and residual muscle channels with no decreasing linear trend (*p* > 0.05, *F*-test). However, SNRs did vary from session to session. The dashed line represents the mean SNR of surface EMG estimated from literature [67, 68]. (b) Boxplots representing the median root mean squared (RMS) of EMG for P1 and P2’s intramuscular electrode channels. Orange lines show the median, blue box shows the interquartile range (IQR), black dashed lines show the most extreme non-outlier values and red crosses show outliers more than 1.5 times the IQR.

To quantify the long-term stability of intramuscular EMG signals over time, participants completed a virtual posture switching task monthly up to 12 and 35 months for participant 1 (P1), and participant 2 (P2), respectively. The SNR of participants P1 and P2’s RPNIs remained consistently high, ranging from 15 to 250 across sessions (figure 2(a)). These SNRs were captured up to 276 and 1054 d post-electrode implantation for P1 and P2, respectively. The median (interquartile range; IQR) SNR of RPNIs was 47.61 (103.89) for P1 and 24.49 (18.53) for P2. Comparatively, the median SNR of residual muscles was 83.72 (111.9) for P1 and 25.23 (40.72) for P2. Interestingly, P1’s RPNIs had a significant linear increase in SNR amplitude over time (*p* < 0.05, *F*-test), whereas P2’s RPNIs did not have significant increasing or decreasing linear trends in SNR amplitudes (*p* = 0.97, 0.12, 0.07 for median RPNI, ulnar RPNI 1, and ulnar RPNI 2, respectively, *F*-test) across sessions.

Though all electrode channels showed high amplitude SNRs on each day, the SNR magnitude varied between sessions. Breaking down the SNR into its numerator (RMS of EMG) and denominator (RMS of electrode noise floor) components, we first investigated if the variability was being driven by the RMS of the electrode noise floor. For both P1 and P2, the RMS of the electrode noise floor across all channels remained stable with a median (IQR) of 1.43 *µ*V (0.44 *µ*V). On the other hand, the EMG RMS range was higher across both RPNIs and residual muscles, with median of 148.2 *µ*V (45.1 *µ*V) for P1 and 39.6 *µ*V (40.5 *µ*V) for P2 (figure 2(b)). Therefore, the variability of SNR was likely driven by the variability of the EMG signal rather than shifts in electrode noise.

### 3.2. Prosthetic grasp classification remains high without recalibration

**Figure 3.**
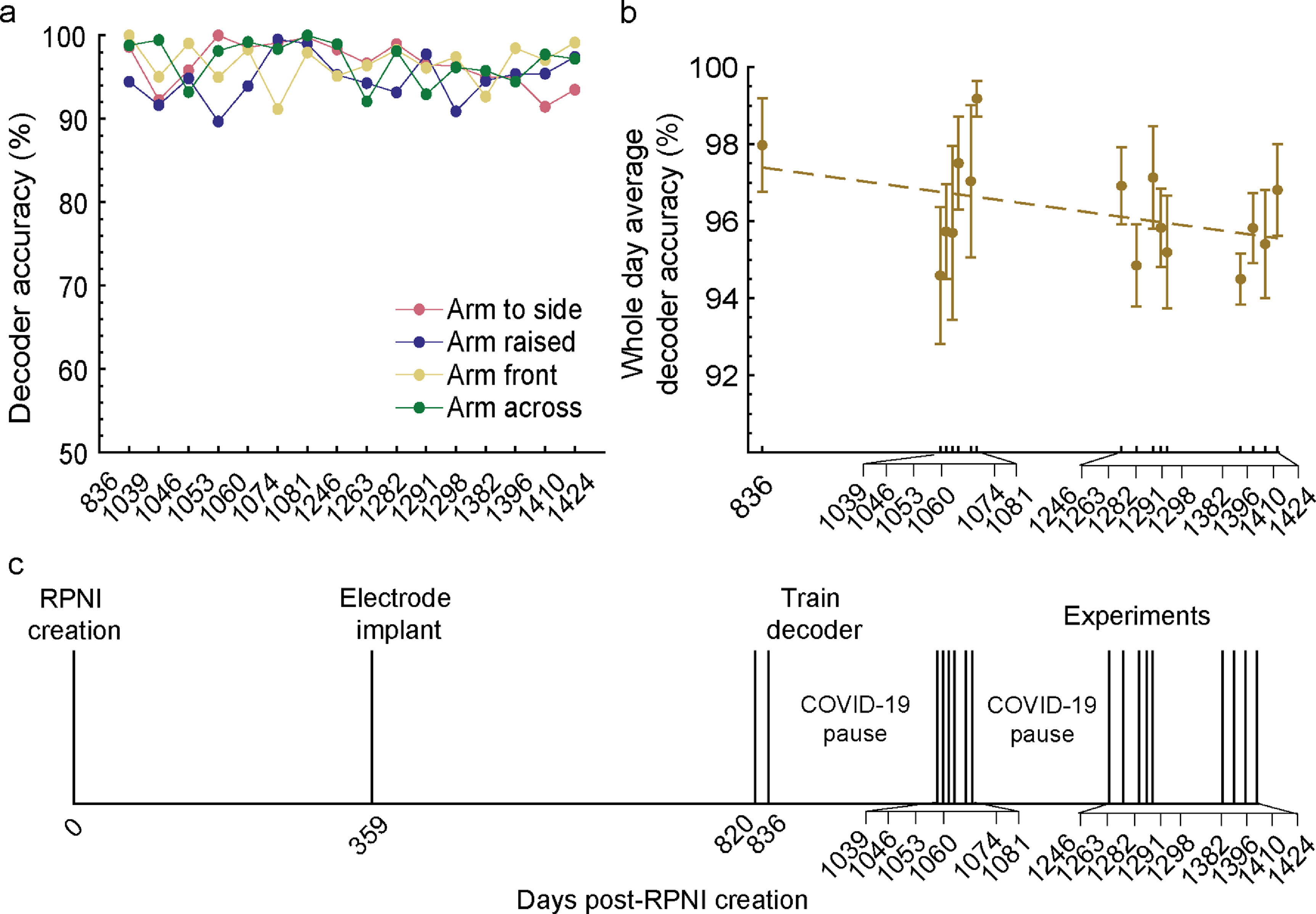
Online four-grip classifier decoding performance across time without recalibration. (a) Decoding accuracy was measured every 50 ms, accounting for transition errors that occurred within each trial. Performance was quantified over four different arm positions: arm at side (blue), arm raised (navy blue), arm front (gold), and arm across (red). (b) Cumulative decoding performance within each session showed no significant linear decreasing performance over time (*p* = 0.11, *F*-test). Gold dashed line represents the linear fit across data points. (c) Timeline of when P2 received RPNIs, electrode implantation, and when the four-grip classifier was trained. Last quantified decoding session occurred 1424 d after RPNI creation.

In a real-world environment, users prefer not to recalibrate their control system every day to achieve high accuracy. To quantify the long-term prosthesis performance using intramuscular signals, we evaluated our participant’s ability to use the same decoder parameters over a period of 16 months. P2 used a four-grip (Rest, Fist, Two-finger pinch, Index finger point) Hidden Markov Model based classifier (HMM-NB) to complete a posture switching virtual task. She completed the task in multiple arm positions without recalibration from the original training session (see methods). Across all sessions, P2 successfully maintained a 1 s hold within a 5-s timeout period on 100% of all trials without recalibration up to 604 d following electrode implantation. Decoder accuracy measured every 50 ms, which accounts for transition errors, remained above 94% with no significant linear decrease in performance across sessions (*p* = 0.11, *F*-test) (figure 3). Across different arm positions, there was a significant difference in the occurrence of transition errors per trial between arm at the side and arm raised (*p* < 0.05, one-way ANOVA with Bonferroni correction for multiple comparisons). The number of transition errors between other postures was not significant (*p* = 0.052 between arm raised and arm front, *p* = 0.38 between arm raised and arm across, *p* = 1.00 between arm to the side, arm front, and arm across).

**Figure 4.**
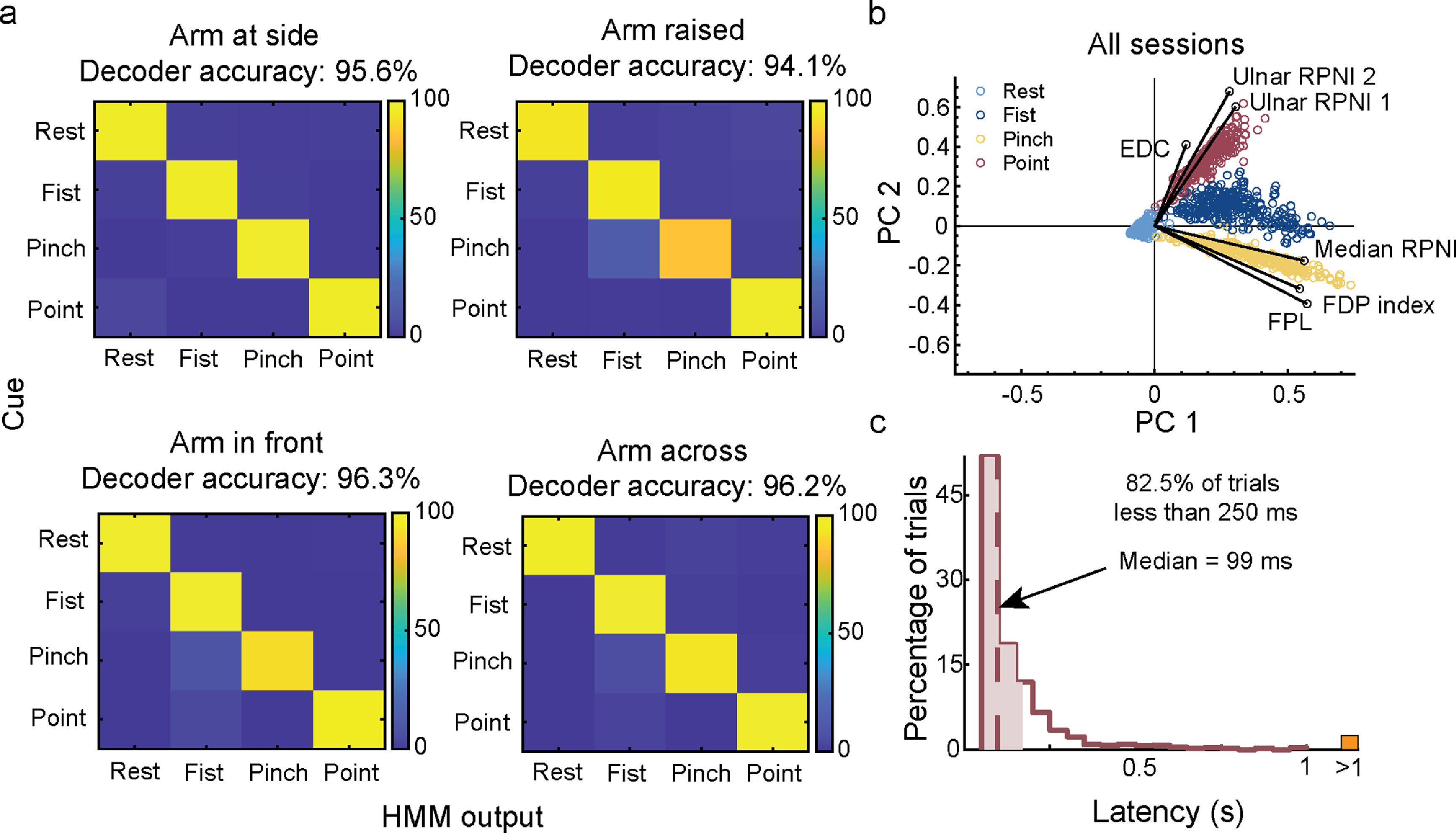
Breakdown of online decoding performance for each arm position across all sessions. (a) Online confusion matrices representing the overall accuracy for each grip (rest, fist, pinch, point) at each arm position (arm at side, arm raised, arm in front, arm across). Confusion matrix captures transition errors to cued grips while P2 controlled the virtual hand in real-time. (b) EMG mean absolute value (MAV) features from both RPNIs, and residual muscles were decomposed into a two-dimensional space for cluster visualization. Cluster separation was seen across all grips: rest (light blue), fist (navy blue), pinch (gold), point (red). Magnitude of the black solid lines represent the contribution each channel provided to each grip. (c) Decoder latency was measured as the time difference between the onset of new EMG activity and a successful posture. The median (dashed line) and middle 50% (shading) is overlaid on histograms binned in 50 ms increments (*n* = 1179 trials). Trials with latency greater than a second (>1) are aggregated in the orange rectangle.

The mean decoder accuracy per timestep across all 16 sessions was 95.6%, 94.1%, 96.3%, and 96.2% for arm at the side, arm raised, arm in front, and arm across, respectively (figure 4(a)). The few grip misclassifications that did occur were between fist and pinch, and fist and point. To better understand the cause for the misclassifications, we decomposed the six-channel EMG inputs into 2D space using ICA with dimensionality reduction using principal component analysis (ICAPCA) to visualize the signal features [69]. This revealed that each grip maintained visually separable clusters across all sessions, but the clusters remained in proximity with one another (figure 4(b)). This analysis is particularly helpful for understanding transition errors for models such as LDA or NB which rely on differences between classes to make decisions. Results also explained why the classifier was able to decode point and pinch with 100% accuracy since they had the greatest cluster separation, but was susceptible to misclassifying between fist and pinch, and fist and point. Confusion between fist and pinch accounted for 41% of transition inaccuracies, and this was most apparent during the arm raised position. Lastly, of the 1673 trials performed, 82.5% of trials had prediction speeds of less than 250 ms from EMG onset, which is well below the 300 ms threshold of perceived delay between muscle activation and prosthetic hand movement [70] (figure 4(c)).

### 3.3. 4-grip classifier translates to real-world task

Next, we determined whether the high decoder classification accuracy in the virtual task translated to real world applications where the participant moved around a physical workspace wearing her prosthesis. Specifically, P2 completed a physical task using the same four-grip controller described above without recalibration. A grip-sequence based activity of daily living was created to demonstrate robustness and intuitive control of the four-grip classifier. We asked P2 to perform a ‘coffee task’ that required grip transitions between fist, pinch, point, and open (finger abduction). P2 was able to switch between each grip to smoothly transition from one segment of the task to the next (movie S1). To quantify grip classification accuracy, P2 was asked to attempt each segment of the coffee task up to five times or until she achieved the correct grip. P2 completed the task with 99% accuracy (*n* = 5 trials, 125 total movements) with only one task error (1 in 125) of dropping the coffee pod. Similarly, grip accuracy was 99% (*n* = 5 trials, 125 movements) with one grasp error of transitioning into a fist instead of a point. From start to finish, P2 completed the coffee task in 61.3 s ± 6.34 on average (*n* = 5 trials). The continuous and segmented coffee task sessions were completed 590 and 611 d post-decoder training, respectively.

### 3.4. Offline nine-grip classifiers decreased in performance without recalibration

**Figure 5.**
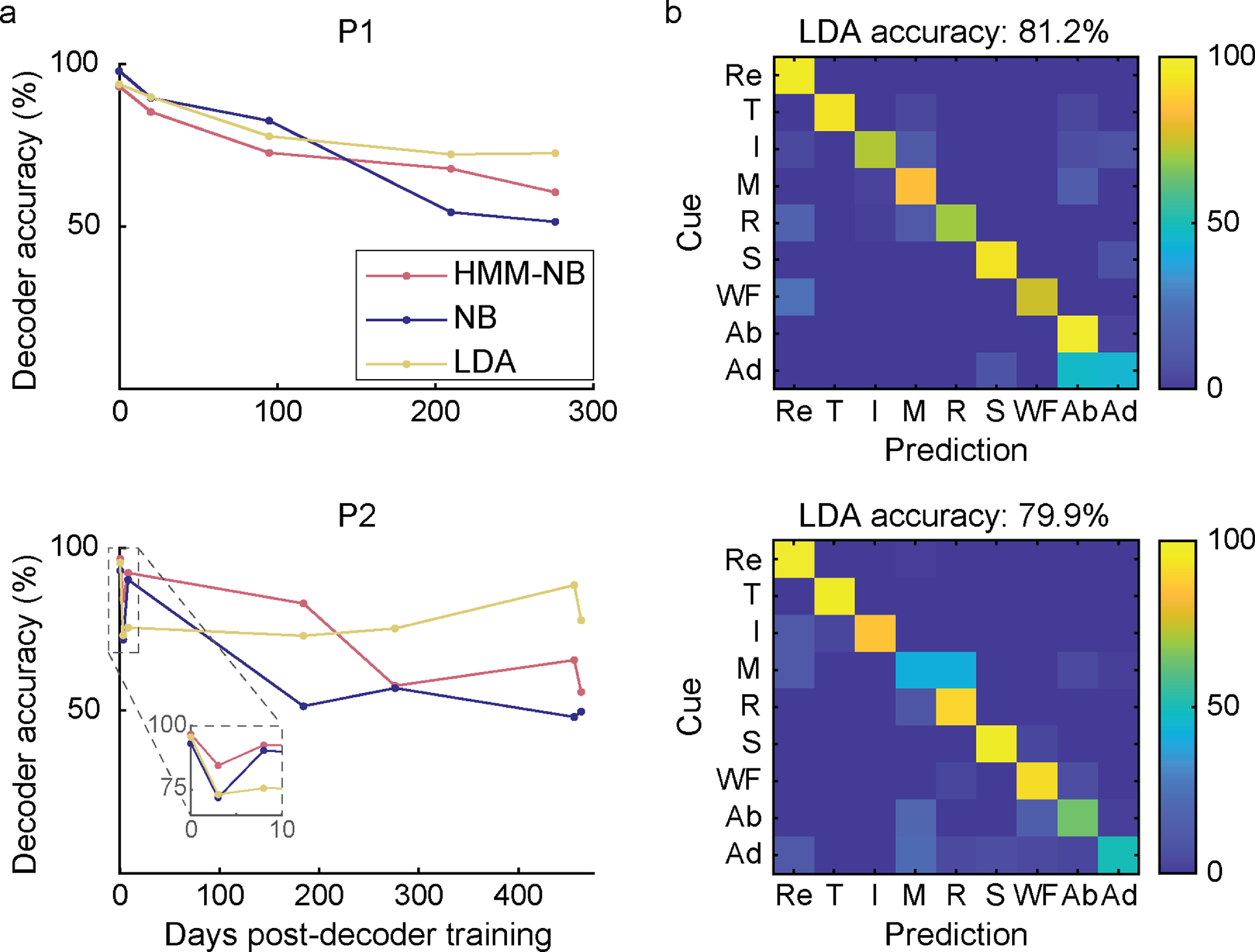
Offline decoding analysis of nine movements over time. (a) Decoding accuracy of Hidden Markov Model (HMM-NB), Naïve Bayes (NB), and Linear Discriminant Analysis (LDA) classifiers over time, without recalibration. Each classifier was trained on nine finger and wrist movements: rest, thumb flexion (T), index finger flexion (I), middle finger flexion (M), ring finger flexion (R), small finger flexion (S), wrist flexion (WF), finger abduction (Ab), and finger adduction (Ad). (b) Offline confusion matrices representing performance of LDA averaged across all sessions (*n* = 5 and 7 sessions for P1 and P2).

Lastly, we wanted to determine how well pattern recognition systems performed without recalibration with more than four movements. In an offline analysis, we trained eight additional movements (thumb flexion (T), index finger flexion (I), middle finger flexion (M), ring finger flexion (R), small finger flexion (S), wrist flexion (WF), finger abduction (Ab), finger adduction (Ad)) and we simulated performance across three different classifiers—HMM-NB, single-state NB, and LDA, in both P1 and P2. The average decoding accuracy across all days post-decoder training was 75.9%, 75.2%, and 81.2% (HMM-NB, NB, LDA, respectively; *n* = 5 sessions) for P1, and 76.5%, 65.9%, and 79.9% for P2 (*n* = 7 sessions). All decoders except P2’s LDA had a small but significant linear decrease in performance across sessions (*p* < 0.05, *F*-test) (figure 5(a)). LDA had the highest average performance across days, suggesting that it may be more robust to changes in EMG activation strength, however the difference was not statistically significant (*p* = 0.220, Wilcoxon rank sum test). Unsurprisingly, these results indicate that predicting an increased number of movements without recalibration may be more challenging. Most classification errors occurred between adjacent postures (abduction and adduction for P1, middle and ring fingers for P2) (figure 5(b)). These movements were either primarily controlled by muscles that were not implanted, or shared ulnar nerve function. Nevertheless, accuracy was greater than 80% for five movements across 276 d for P1, and six movements across 463 d for P2.

### 3.5. RPNIs contribute to the prediction of intrinsic finger movements

**Figure 6.**
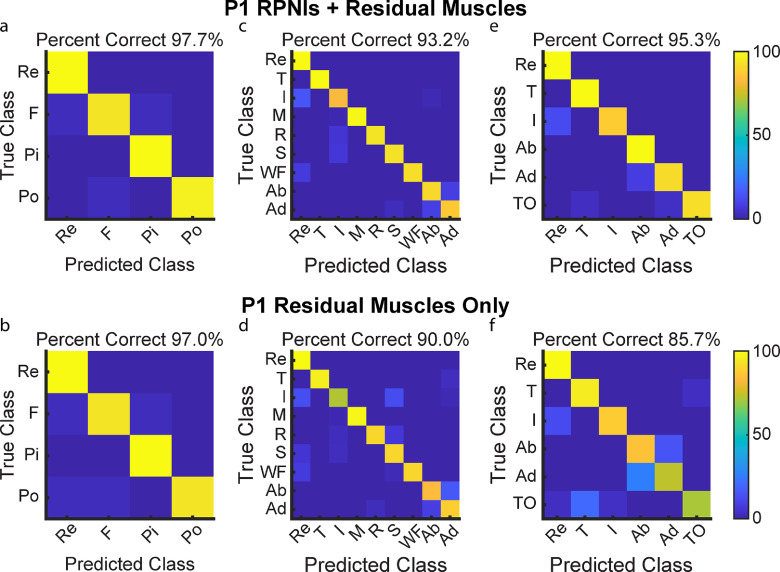
P1 offline decoding performance with RPNIs and residual muscles vs. only residual muscles. (a), (b) Confusion matrices show a Linear Discriminant Analysis (LDA) classifier predicting four movements for grasp control: fist (F), pinch (Pi), point (Po), and rest (Re) with and without input from P1’s RPNIs. (c), (d) Prediction performance of the individual finger movements: rest, thumb flexion (T), index finger flexion (I), middle finger flexion (M), ring finger flexion (R), small finger flexion (S), wrist flexion (WF), finger abduction (Ab), and finger adduction (Ad), with and without RPNIs. (e), (f) Distinguishing rest and index finger flexion from four thumb and intrinsic finger movements: thumb flexion, finger abduction, finger adduction, and thumb opposition (TO), with and without RPNIs.

**Figure 7.**
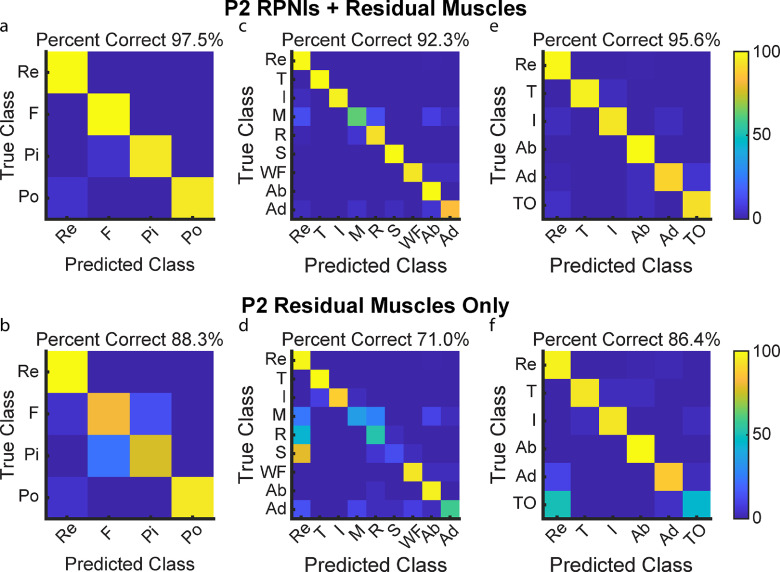
P2 offline decoding performance with RPNIs and residual muscles vs. only residual muscles. (a), (b) Confusion matrices show a Linear Discriminant Analysis (LDA) classifier predicting the four movements for grasp control with and without RPNIs. (c), (d) Prediction performance of the nine individual finger movements analyzed with and without RPNIs. (e), (f) Distinguishing rest and index finger flexion from the four thumb and intrinsic finger movements with and without P2’s RPNIs.

P1 and P2 both had transradial amputations and electrodes implanted into six and five residual innervated finger and wrist muscles which were used for movement decoding in addition to their RPNIs. To understand the contribution of RPNIs to finger and grasp prediction, we compared the performance of movement classifiers offline to see which movements were most impacted without input from RPNIs. For P1, grip accuracy only decreased by 0.7% when removing RPNI signals, indicating his implanted residual muscles may be sufficient to control those movements (figures 6(a) and (b)). Removing RPNIs decreased overall accuracy by 3.2% for the nine movement dataset (figures 6(c) and (d)), and 9.6% for an intrinsic movement dataset (figures 6(e) and (f)). For P2, grip accuracy decreased by 9.2% (figures 7(a) and (b)), accuracy of the nine movement dataset decreased by 21.3% (figures 7(c) and (d)), and accuracy of the intrinsic movement dataset decreased by 9.2% (figures 7(e) and (f)) when RPNIs were removed. In both patients, RPNIs most strikingly contributed to accurate prediction of thumb opposition while also improving the distinction of finger adduction. For P2, RPNIs also greatly improved prediction of middle, ring, and small finger movements as well as distinguishing fist from pinch. These movements are all either controlled by lost intrinsic hand muscles or extrinsic muscles that were not implanted (for instance, FDP small finger was implanted for P1 but not P2). These results are consistent with recent online control comparisons [71], and indicate that RPNIs provide valuable control signals when muscles are lost due to amputation.

## 4. Discussion

Developing a reliable and intuitive upper-limb prosthetic control system has been the goal of clinicians, engineers, and prosthetic end-users for decades [15, 72–74]. Without a stable peripheral nerve interface, the potential of controlling multi-articulated prosthetic hands will be difficult to realize for people with upper-extremity limb loss. Here, we have demonstrated that intramuscular electrodes embedded within RPNIs, and residual innervated muscles can produce high amplitude EMG signals across multiple years in individuals with amputation. The process of free muscle graft regeneration, revascularization, and reinnervation has been established for many years [75–77]. Expanding upon this foundational knowledge, RPNIs utilize a free skeletal muscle graft which is neurotized by a transected peripheral nerve or nerve fascicle. As detailed by Srinivasan *et al*, the skeletal muscle graft survives through a series of biologic processes identical to those seen with skin graft survival: (1) plasmatic imbibition; (2) inosculation; (3) capillary ingrowth, followed by; (4) formation of large blood vessels [78]. Subsequently, the healthy skeletal muscle grafts are reinnervated after the implanted peripheral nerve undergoes axonal sprouting, elongation, and neuromuscular junctions (NMJs) formation to create the functional RPNI. The RPNI can then act as a bioamplifier of efferent motor action potentials allowing large EMG signals to be recorded from the RPNI rather than small signals recorded directly from the peripheral nerve.

Though we recorded reliably large signals from the muscle grafts and residual innervated muscles, the EMG amplitudes did vary substantially from day to day, even with nominally consistent attempted movements. While there may be some position shifting effect from the electrodes, we did not observe any substantial cluster centroid deviation across arm positions as commonly seen with surface EMG [38]. Thus, day-to-day variability in the EMG may instead reflect natural variation when attempting the same movement across days during a task that provides no visual feedback. Similar results have been reported for individuals without amputation making movements over time [79]. This creates a challenge for designing robust classifiers, and more training data may ultimately be required for algorithms to learn the full range of possibilities. Techniques developed for multi-day surface EMG may also be applicable with intramuscular signals [80, 81]. We may expect that signals that are reliably high and under voluntary control would become intuitive to use with closed loop control. As expected, one of our participants who used one decoder across 1.7 years can don and immediately begin using the prosthesis with similar success from day to day in the lab despite showing an EMG interquartile range of 40.5 *µ*V. This likely reflects both an intuitive use of the correct muscles and consolidated learning from the participant, who had previously completed grasp control tasks with the implanted electrodes for another study [63]. Further studies may show that learning is much improved with a stable relationship to voluntary EMG from day to day.

While multi-grasp pattern recognition systems have been around for decades [82] with the first commercial system launched in 2015 (Co-Apt, IBT, Ottobock), clinical application of these systems have been challenging. In particular, offline analysis has shown performance degradation over time caused by surface electrode shifting or changes in residual limb position [38, 83, 84]. In order to improve pattern recognition decoding performance, complex surface EMG training paradigms, such as recording EMG at different arm positions, have been developed [85–88]. However, these paradigms may be impractical in commercial prosthetic control, as the offline assessments conducted within laboratory conditions may not fully reflect the functional efficacy of pattern recognition systems in real-world scenarios [89]. A recent take-home study highlights this challenge, as the users preferred pattern recognition over direct control for intuitive switching of hand postures but had to recalibrate their control signals an average of 33 times during a 148 hour wear time [29]. Our study found that intramuscular EMG recorded from reinnervated and residual muscles provides high enough specificity and SNR despite changes in absolute voltage levels to maintain feature separation for consistent prosthetic performance over time without recalibration. Importantly, the HMM-NB was trained only with five trials of each grasp, while the participant sat motionless in front of a computer screen. Minimal, or possibly zero recalibrations using a simple decoder training protocol may be a desirable characteristic for upper-limb prosthetic control systems to improve satisfaction and reduce prosthetic abandonment among prosthesis users [90].

Ultimately, regardless of the surgical preparation or algorithm used, the large SNR generated from intramuscular electrodes has proven to be valuable for better prosthetic control. By comparison, the SNR from surface EMG is typically in the range of 2–20 and varies depending on tissue thickness and the use of dry vs. gelled adhesive electrodes [67, 68, 91]. There are large efforts already underway to better interpret surface EMG [92–94]. Algorithms developed using surface EMG will likely perform better, or at minimum more reliably, when used with implantable EMG. Simultaneously, the development of more advanced prosthetic hands with added hardware features such as additional DoFs at the thumb and wrist [95, 96] has the potential to reduce compensatory movements and prevent overuse injuries. Overall, our study has demonstrated the long-term robustness of RPNIs as a peripheral nerve interface. For patients with transradial amputations, the best performance can be achieved with a combination of EMG from extrinsic residual muscles and EMG from RPNIs as was done in this study. We observed the RPNIs contributed most to predicting thumb and finger movements controlled by lost intrinsic hand muscles or extrinsic muscles that were not implanted. Patients with more proximal amputations may be missing most or all of their finger or wrist muscles. In these cases, we expect RPNIs with implanted electrodes would provide necessary signals for accurate and reliable control of multiple hand and wrist functions. With these promising preliminary results, implantable prosthetic hand controllers may soon become the standard of care for all upper limb amputations.

## Data Availability

The data that support the findings of this study are available upon reasonable request from the authors.

## Materials and correspondence

Materials and correspondence requests should be addressed to P P V.
